# The efficacy and safety of moxibustion for chronic fatigue syndrome: A protocol for systematic review and meta-analysis

**DOI:** 10.1097/MD.0000000000025742

**Published:** 2021-05-07

**Authors:** Kaiyang Xue, Yanping Wang, Xianzhu Wang, Pingnan Chen, Caihong Xiao, Jing Fu, Jin Cui

**Affiliations:** a Guizhou University of Traditional Chinese Medicine, Guizhou; b Jiangxi University of Traditional Chinese Medicine, Jiangxi, China.

**Keywords:** chronic fatigue syndrome, meta-analysis, moxibustion, protocol, systematic review

## Abstract

**Background:**

The pathogenesis of chronic fatigue syndrome (CFS) is not clear. The main purpose of treatment is to improve autoimmune function and relieve fatigue symptoms. Moxibustion is often used to treat diseases caused by low autoimmunity, especially in relieving fatigue symptoms. It is a superior therapy for CFS in traditional Chinese medicine. At present, there is a lack of the high level clinical evidence to support the moxibustion in the treatment of CFS, so this study will systematically review and analyze the currently available randomized controlled trials to evaluate the efficacy and safety of moxibustion in the treatment of CFS.

**Methods:**

We will systematically search PubMed, EMBASE, Cochrane library, Sinomed, CNKI, VIP, and Wanfang Database, ClinicalTrials.gov and Chinese Clinical Trial Registry will also be searched. The time range for the search will be from database activation to March 31, 2021. The randomized controlled trials (RCTs) associated with moxibustion for CFS will be included, regardless of language.

We will use the standard proposed in Cochrane Handbook 5.1.0 to assess the bias risk of a single RCT. The main outcome index of the study is Fatigue Assessment Instrument (FAI), secondary outcome indexes will include Fatigue Scale -14 (FS-14), Fatigue Severity Scale (FSS), Pittsburgh sleep quality index (PSQI), natural killer (NK) cells, interleukin- 2 (IL-2), T lymphocyte subsets (CD_4_^+^, CD_8_^+^), cure rate, total efficiency and adverse reactions. The random effect model meta was used to analyze the effect data of a single RCT. Heterogeneity will be measured by Cochran Q test and *I*-squared statistics. We will use 2 subgroup analyses to explore the source of heterogeneity. RCTs with high bias risk was excluded and adjustment effect model was used for sensitivity analysis to test the robustness of the meta-analysis results. The publication bias included in RCTs will be assessed by funnel plot and Egger test.

**Results:**

This study will objectively and comprehensively evaluate the efficacy and safety of randomized controlled trials of moxibustion in the treatment of chronic fatigue syndrome, and the results will be submitted to peer-reviewed journals for publication.

**Conclusion:**

This systematic review will provide clinicians with the latest high-quality evidence for the use of moxibustion in the treatment of chronic fatigue syndrome.

**INPLASY registration number:**

INPLASY202140063.

## 1 Introduction

### 1.1 Description of the condition

Chronic fatigue syndrome is a worldwide disease of unknown cause, which is characterized by long-term extreme fatigue for more than 6 months, accompanied by inattention, memory loss, sleep disorders, and many other nonspecific symptoms.^[1]^ Chronic fatigue significantly reduces the quality of patients’ life and even may induce dysfunction, which has become one of the most severe public health problems in the world.^[2]^ A recent systematic review showed that the prevalence of CFS ranges from 0.01% to 7.62%, and the prevalence of women is 1.5 to 2 times higher than that of men.^[3]^ A previous study found that only 13% of CFS patients were able to stick to their jobs, more than 25% of CFS patients were forced to be isolated at home, and severe cases were bedridden.^[4]^ At present, the etiology and pathogenesis of CFS are not yet clear, and the neuro-endocrine-immune network may be the key link in the pathogenesis of CFS.^[5]^ Modern medicine mainly supports the treatment of CFS such as anti-anxiety, immune regulation, and nutritional supplementation, but the effectiveness is still controversial.^[6–8]^

### 1.2 Description of the intervention

The origin of moxibustion can be traced back to primitive society.^[9]^ It is an external treatment method that burns moxa, produces moxa smoke, and applies heat to acupoints or lesions on the body surface to prevent and cure diseases.^[10]^ Moxa is the most commonly used moxibustion material. Burning of moxa has the effect of warming yang and dispelling cold, activating meridians, and strengthening the body, especially suitable for chronic weakness diseases such as CFS. Moxibustion can be divided into direct moxibustion that burns moxa directly above the skin,^[11]^ and indirect moxibustion that separates media between moxa and the skin (such as ginger, garlic, salt, medicinal cake, etc).^[12]^ Indirect moxibustion combines the effects of moxa and the medium, and the synergistic effect can be achieved by transdermal administration of moxa heat. Studies have shown that moxibustion can effectively relieve fatigue,^[13,14]^ and improve sleep quality,^[15]^ pain and paresthesia,^[16]^ gastrointestinal dysfunction,^[17]^ and other CFS-related symptoms.

### 1.3 How the intervention might work

The mechanism of moxibustion in the treatment of chronic fatigue syndrome may be related to neuroendocrine, immune inflammation, energy metabolism and so on. Yi et al found that moxibustion can increase the expression of procorticotropin mRNA and protein in the hippocampus, and relieve the fatigue symptoms of CFS rats by inhibiting the excessive activity of the hypothalamus-pituitary-adrenal axis.^[18]^ Studies have shown that the proinflammatory cytokine IL-6 may be an essential factor in inducing fatigue.^[19]^ Several teams have proposed that moxibustion can regulate the levels of peripheral and central proinflammatory cytokine IL-6 and anti-inflammatory cytokine IL −10 to help rats recover from fatigue.^[20–22]^ Moxibustion can regulate the energy metabolism of rats with exercise-induced fatigue. Lu et al found that moxibustion can increase the liver glycogen content of exhausted fatigue rats.^[23]^ Gao et al also confirmed that moxibustion can significantly up-regulate the glycogen content of the gastrocnemius muscle of fatigued rats, reduced the level of lactic acid, and delayed the occurrence of exercise-induced fatigue.^[24]^

### 1.4 Why it is important to perform this review

Moxibustion does not invade the body, nor does it need to bear the toxic and side effects of drug metabolism. It is one of the most popular treatment methods for patients. However, due to insufficient evidence of clinical efficacy, as another important part of acupuncture therapy in addition to “needle,” “moxibustion” is not as popular as “needle therapy” in both Eastern and Western countries, people often mistakenly believe that acupuncture is just a treatment method about needling. Some systematic reviews and meta-analyses combine moxibustion and needle therapy as a whole, but there is few systematic review and meta-analysis for moxibustion alone. With the publication of more and more randomized controlled trials of moxibustion in the treatment of CFS, it is necessary to use comprehensive and accurate retrieval methods to evaluate the effectiveness and safety of moxibustion in the treatment of CFS, which will help clinical doctors, researchers and patients to understand the safety and efficacy of moxibustion in the treatment of CFS, providing evidence support for clinical decision-making.

### 1.5 Objective

The efficacy and safety of moxibustion in the treatment of CFS will be evaluated by systematically reviewing and analyzing the currently available randomized controlled trials.

## 2 Methods

### 2.1 Study registration

The program was registered on the INPLASY platform (Registration Number:INPLASY202140063, https://inplasy.com/inplasy-2021-4-0063). We reported the program according to the preferred reporting program of the systematic review and meta-analysis program (PRISMA-P).^[25]^

### 2.2 Inclusion and exclusion criteria

#### 2.2.1 Type of study

We will include the randomized controlled trials of moxibustion in the treatment of CFS, whether published or not. However, case reports and comments, clinical studies comparing different types of moxibustion, randomized crossover trials, animal trials, and the literature only published in the form of abstracts will be excluded. For republished research, the RCT with the latest results and the longest follow-up period will be selected.

#### 2.2.2 Participants

We will include patients according to the criteria of the US CDC definition (1988^[26]^ or 1994^[1]^) for CFS, without restrictions on gender, age, course of disease, severity, and source of cases.

#### 2.2.3 Interventions and controls

Moxibustion that ignites moxa and produces moxa smoke and temperature is the intervention measure concerned in this study. We do not restrict the production forms of moxa (moxa cone, moxaroll), nor the types of moxibustion (direct moxibustion, indirect moxibustion), but electronic moxibustion will be excluded. We will include the following 2 types of randomized controlled trials:

1. The experimental group uses moxibustion alone to treat CFS, and the control group uses other methods except moxibustion to treat CFS;
2. The experimental group uses moxibustion combined with other methods to treat CFS, and the control group uses the same combined method alone as the experimental group to treat CFS.

#### 2.2.4 Outcomes

##### 2.2.4.1 Primary outcomes

The main outcome is Fatigue Assessment Instrument (FAI).^[27]^

##### 2.2.4.2 Secondary outcomes

Secondary outcome: Fatigue Scale-14 (FS-14), Fatigue Severity Scale (FSS), Pittsburgh Sleep Quality Index (PSQI), natural killer (NK) Cells, interleukin 2 (IL-2), T lymphocyte subsets (CD_4_^+^, CD_8_^+^), cure rate, total efficiency, and adverse reactions.

### 2.3 Data sources and search strategy

We will systematically search 7 major databases [PubMed, EMBASE, Cochrane library, Sinomed, CNKI (China National Knowledge Infrastructure), VIP (Chinese Scientific Journals Database) and Database Wanfang] and collect the RCTs of moxibustion treatment of CFS. The time range of the search will be from the database to March 31, 2021. We will use a combination of medical subject words and free words to search for terms such as “Chronic Fatigue syndrome,” “chronic Fatigue,” “and Moxibustion,” a search strategy in PubMed was listed in Table 1. For including more studies, we will also search the 2 trial registration platforms ClinicalTrials.gov and Chinese Clinic Trials.gov, as well as references from related reviews.

**Table 1:** Search strategy in PubMed.

No.	Search terms
1	chronic fatigue syndrome[mh]
2	chronic fatigue syndrome[tw]
3	chronic fatigue [tw]
4	fatigue syndrome[tw]
5	fatigue disorder[tw]
6	fatigue fibromyalgia syndrome[tw]
7	myalgic encephalomyelitis[tw]
8	#1 OR #2 OR #3 OR #4OR #5OR #6OR #7
9	moxibustion[tw]
10	moxibustion therapy [tw]
11	#9 OR #10
12	animals[mh]
13	humans[mh]
14	#8 AND #11
15	#12 NOT #13
16	#14 NOT #15

### 2.4 Data collection and analysis

#### 2.4.1 Study selection

**Figure 1:**
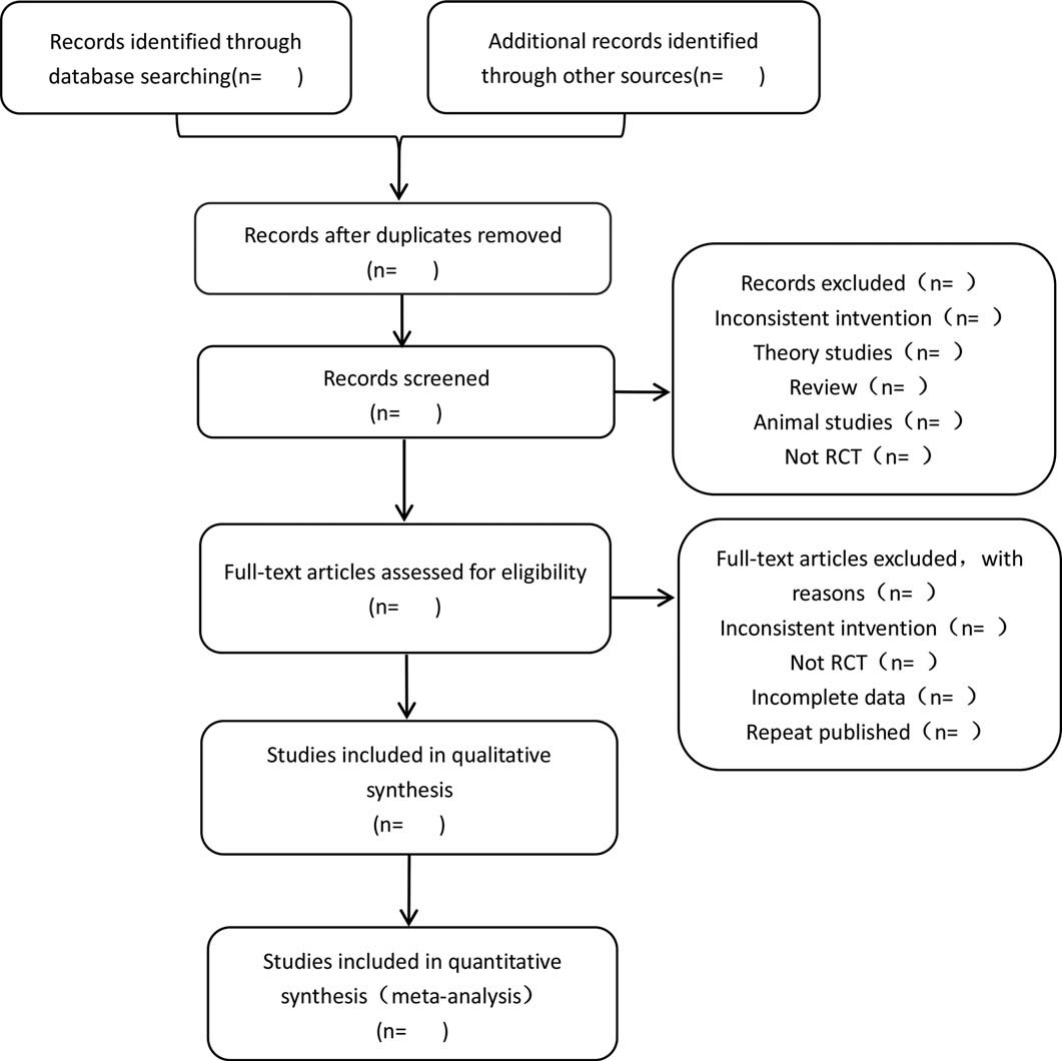
PRISMA-style flow chart of literature screening.

The retrieved research literature will be imported into Endnote X9 (clarivate analytics US LLC) to exclude repetitive literature. Two reviewers will screen the literature independently and repeatedly. Firstly, irrelevant literature will be excluded by reading the title and abstract, and then the full text will be further checked to determine whether to include it. The reviewers will cross-check the results, they will return to the screen together when there is difference, and consult the third reviewer to resolve them if necessary. The flow chart of APRISMA-style literature screening is shown in Figure 1.

#### 2.4.2 Data extraction

We will use the standardized tables to extract data from included RCTs. It consists of 3 parts group to:

1. Publishing features: title, first author, establishments, journals, and publication date;
2. patients and treatment: sex, age, source of the patients, sample size, with or without blinding, moxibustion site, moxibustion strength, duration of treatment, adverse reactions, adverse events, follow-up time, loss rate, and reasons;
3. outcome data: baseline and follow-up measurement of each index.

#### 2.4.3 Risk of bias assessment

The risk of bias included in the RCTs will be obtained using the risk of bias assessment tool in the Cochrane Handbook 5.1.0.^[28]^ It includes 7 projects:

1. random sequence generation method,
2. allocation concealment method,
3. blinding method of clinicians and patients,
4. blinding method of result evaluators,
5. completeness of data,
6. selective reporting, and
7. other sources of bias.

Items 1 and 2 indicates selection bias, while items 3, 4, 5, and 6 which reflects performance bias, detection bias, loss of follow-up bias, and reporting bias, respectively. According to the description of the method in each study, each will be rated as a low, high, or uncertain risk. Two examiners will independently and repeatedly assess the bias and cross-checks. Any difference will be resolved through discussions with the third examiner.

#### 2.4.4 Managing missing data

We will try to obtain exact information of any missing data in the full text of the RCT by sending an email to the corresponding author. If the author can not provide the missing standard deviation, the median of the standard deviation in other randomized controlled trials will be used to estimate the missing standard deviation.

#### 2.4.5 Data synthesis

We will extract data from individual randomized controlled trials through RevMan version 5.3 meta-analysis of random effects model (Copenhagen: Nordic Cochrane Center, Cochrane Collaborative Organization, 2014). According to its normality, the weighted mean deviations (WMDs) or standardized mean deviations (SMD) will be used to measure the impact on continuous results, and the inverse variance method will be used as a meta-analysis method. Using the mantel Haenszel method, risk ratio (ORS) will be used to measure the result of binary classification. 95% confidence interval (CIS) will be calculated to express the estimation accuracy.

#### 2.4.6 Assessment of heterogeneity

In the meta-analysis, we will use the Cochran Q test to determine whether there is statistically significant heterogeneity among randomized controlled trials, and use the I-squared statistic to quantitatively detect the level of heterogeneity. When a *P* value lower than .10 or an *I*^2^ higher than 50%, there is heterogeneity among the studies, and the source of the heterogeneity should be analyzed first. If the source of heterogeneity is obvious, such as obvious abnormalities in the original data, large differences in the quality of the original research and treatment time, etc. Sensitivity analysis or subgroup analysis according to the sources of differences should be performed.

#### 2.4.7 Subgroup analysis

Based on the experience of moxibustion therapy in clinical application, when there is obvious heterogeneity in the analysis results, we will analyze the source of the heterogeneity from the following subgroups:

1. Subgroup analysis by moxibustion type: the randomized controlled trial of indirect moxibustion was compared with that of direct moxibustion. It is expected that the former has a better curative effect.
2. Subgroup analysis by treatment course: we will compare randomized controlled trials with a course of treatment >2 weeks and ≤2 weeks. The former is expected to have a better effect.

#### 2.4.8 Sensitivity analysis

In order to verify the robustness of the meta-analysis results, we will conduct the following 2 sets of sensitivity analyses:

1. excluding studies with high risk of bias;
2. using a fixed-effects model to collect the data from the meta- analysis.

#### 2.4.9 Assessment of publication bias

We will draw a funnel chart and perform an Egger test to determine whether there is a potential publication bias in the case of a sufficient number of studies. The funnel chart is relatively symmetrical, when there is no bias in the analysis results. If the distribution pattern is asymmetric, it can be considered that there is publication bias, and further screening is needed.

#### 2.4.10 Assessment of the quality of evidence

We will assess the quality of evidence for each research result based on the Grading of Recommendations Assessment, Development and Evaluation (GRADE) framework. We rate the quality of evidence in 5 areas (risk of bias, indirection, inconsistency, imprecision, and publication bias). Finally, the quality of evidence will be determined as high (no obvious restrictions in all aspects), medium (downgrade 1), low quality (downgrade 2), and very low quality (downgrade 3 or more).

### 2.5 Ethics and dissemination

This study does not involve personal and human test data, so ethical approval is not required. Our goal is to publish the results of this systematic review in a peer-reviewed journal.

## 3 Discussion

Most of the existing systematic reviews are based on the combination of needle therapy and moxibustion, but there is few systematic review with high-quality of moxibustion in the treatment of chronic fatigue syndrome alone. A previous Chinese systematic review evaluated the effectiveness and safety of moxibustion in the treatment of chronic fatigue syndrome,^[29]^ but its methodological quality was low, and the evaluation results were not convincing. The emergence of more convincing randomized controlled trials has promoted the formulation of this systematic review program.

In this systematic review, we will make effort to improve the reliability of the results, including systematic literature search, strict risk of bias, reasonable data analysis, objective evidence evaluation quality, and conduct research in strict accordance with the agreement. We believe that the results of this systematic review will help to improve the evidence of clinical moxibustion in the treatment of CFS for clinical decision-making.

## Author contributions

**Conceptualization:** Jing Fu.

**Funding acquisition:** Jin Cui.

**Investigation:** Xianzhu Wang, Pingnan Chen, Caihong Xiao.

**Methodology:** Kaiyang Xue, Yanping Wang.

**Writing – original draft:** Kaiyang Xue, Yanping Wang, Jing Fu.

**Writing – review & editing:** Jing Fu, Jin Cui.

## References

[1] Fukuda K Straus SE Hickie I. The chronic fatigue syndrome: a comprehensive approach to its definition and study. International Chronic Fatigue Syndrome Study Group. Ann Intern Med 1994;121:953–9. 7978722 10.7326/0003-4819-121-12-199412150-00009

[2] Tomas C Brown A Strassheim V. Cellular bioenergetics is impaired in patients with chronic fatigue syndrome. Plos One 2017;12:e186802. 10.1371/journal.pone.0186802 PMC5655451 29065167

[3] Lim EJ Ahn YC Jang ES. Systematic review and meta-analysis of the prevalence of chronic fatigue syndrome/myalgic encephalomyelitis (CFS/ME). J Transl Med 2020;18:100. 32093722 10.1186/s12967-020-02269-0 PMC7038594

[4] Pendergrast T Brown A Sunnquist M. Housebound versus nonhousebound patients with myalgic encephalomyelitis and chronic fatigue syndrome. Chronic Illn 2016;12:292–307. 27127189 10.1177/1742395316644770 PMC5464362

[5] Cortes RM Mastronardi C Silva-Aldana CT. Myalgic encephalomyelitis/chronic fatigue syndrome: a comprehensive review. Diagnostics (Basel) 2019;9:91. 10.3390/diagnostics9030091 PMC6787585 31394725

[6] Smith ME Haney E McDonagh M. Treatment of myalgic encephalomyelitis/chronic fatigue syndrome: a systematic review for a national institutes of health pathways to prevention workshop. Ann Intern Med 2015;162:841–50. 26075755 10.7326/M15-0114

[7] Bruun WV Bjorneklett A Brubakk O. Diagnosis and Treatment of Chronic Fatigue Syndrome/Myalgic Encephalopathy (CFS/ME). 2006;Oslo, Norway: Knowledge Centre for the Health Services at The Norwegian Institute of Public Health (NIPH), 13–4. 29320016

[8] Reid S Chalder T Cleare A. Chronic fatigue syndrome. BMJ Clin Evid 2000;320:292–6.

[9] Huang C Liang J Han L. Moxibustion in early Chinese medicine and its relation to the origin of meridians: a study on the unearthed literatures. Evid Based Complement Alternat Med 2017;2017:8242136. 28298936 10.1155/2017/8242136 PMC5337347

[10] Chiu JH. How does moxibustion possibly work? Evid Based Complement Alternat Med 2013;2013:198584. 23606872 10.1155/2013/198584 PMC3623111

[11] Thorne TL Hanes DA Wild H. Direct moxibustion to treat spleen qi and yang deficiency fatigue: a pilot study. J Acupunct Meridian Stud 2014;7:76–82. 24745866 10.1016/j.jams.2013.04.003

[12] Kim HG Yoo SR Park HJ. Indirect moxibustion (CV4 and CV8) ameliorates chronic fatigue: a randomized, double-blind, controlled study. J Altern Complement Med 2013;19:134–40. 22757691 10.1089/acm.2011.0503 PMC3576895

[13] Xu HQ Zhang HR Gu YH. Progress of researches on prevention and treatment of sports fatigue with moxibustion therapy. Zhen Ci Yan Jiu 2014;39:169–73. 24818504

[14] Iravani S Cai L Ha L. Moxibustion at ’Danzhong’ (RN17) and ’Guanyuan’ (RN4) for fatigue symptom in patients with depression: Study protocol clinical trial (SPIRIT Compliant). Medicine (Baltimore) 2020;99:e19197. 32049857 10.1097/MD.0000000000019197 PMC7035006

[15] Sun YJ Yuan JM Yang ZM. Effectiveness and safety of moxibustion for primary insomnia: a systematic review and meta-analysis. BMC Complement Altern Med 2016;16:217. 27411310 10.1186/s12906-016-1179-9 PMC4944240

[16] Lee MS Choi TY Kang JW. Moxibustion for treating pain: a systematic review. Am J Chin Med 2010;38:829–38. 20821815 10.1142/S0192415X10008275

[17] Tang B Zhang J Yang Z. Moxibustion for diarrhea-predominant irritable bowel syndrome: a systematic review and meta-analysis of randomized controlled trials. Evid Based Complement Alternat Med 2016;2016:5105108. 27293460 10.1155/2016/5105108 PMC4884811

[18] Yi T Qi L Li J. Moxibustion upregulates hippocampal progranulin expression. Neural Regen Res 2016;11:610–6. 27212922 10.4103/1673-5374.180746 PMC4870918

[19] Robson-Ansley PJ de Milander L Collins M. Acute interleukin-6 administration impairs athletic performance in healthy, trained male runners. Can J Appl Physiol 2004;29:411–8. 15317982 10.1139/h04-026

[20] Zhao Y Li TG Pu R. Effect of moxibustion on body weight and peripheral and cerebral cortical IL-6 and IL-10 levels in fatigue rats. Zhen Ci Yan Jiu 2020;45:215–9. 32202713 10.13702/j.1000-0607.190815

[21] Li TG Pu R Shui L. Effect of moxibustion on inflammatory cytokine levels in hippocampus and hypothalamus in rats with fatigue. Zhen Ci Yan Jiu 2019;44:195–9. 30945502 10.13702/j.1000-0607.180619

[22] Li TG Shui L Ge DY. Moxibustion reduces inflammatory response in the hippocampus of a chronic exercise-induced fatigue rat. Front Integr Neurosci 2019;13:48. 31616260 10.3389/fnint.2019.00048 PMC6763602

[23] Lu J Ge GL Zhang HL. Effect of moxibustion on hepatic glycogen and ultrastructure of exercise-induced fatigue rats. Zhen Ci Yan Jiu 2011;36:32–5. 21585056

[24] Gao M Yang HY Liu TY. Effects of different acupuncture and moxibustion methods on ultrastructure of gastrocnemius in rats. Zhongguo Zhen Jiu 2014;34:261–5. 24843969

[25] Moher D Shamseer L Clarke M. Preferred reporting items for systematic review and meta-analysis protocols (PRISMA-P) 2015 statement. Syst Rev 2015;4:01. 10.1186/2046-4053-4-1 PMC4320440 25554246

[26] Holmes GP Kaplan JE Gantz NM. Chronic fatigue syndrome: a working case definition. Ann Intern Med 1988;108:387–9. 2829679 10.7326/0003-4819-108-3-387

[27] Schwartz JE Jandorf L Krupp LB. The measurement of fatigue: a new instrument. J Psychosom Res 1993;37:753–62. 8229906 10.1016/0022-3999(93)90104-n

[28] Guyatt GH Oxman AD Vist G. GRADE guidelines: 4. Rating the quality of evidence--study limitations (risk of bias). J Clin Epidemiol 2011;64:407–15. 21247734 10.1016/j.jclinepi.2010.07.017

[29] Zhang Y Chen GQ Yan J. Systematic review of moxibustion treatment of chronic fatigue syndrome. J External Therap Tradit Chin Med 2017;26:57–9.

